# Thoracolumbar fractures patients undergoing posterior pedicle screw fixation can benefit from drainage

**DOI:** 10.1186/s12891-024-07447-5

**Published:** 2024-05-01

**Authors:** Jing-yu Sun, Ning Zhao, Hua Chen, Chun-hui Chen

**Affiliations:** Department of Orthopaedic Surgery, The Second Affiliated Hospital and Yuying Children’s Hospital of Wenzhou Medical University, 109 Xueyuanxi Road , Wenzhou, Zhejiang 325000 China

**Keywords:** Thoracolumbar fracture, Pedicle screw fixation, Drainage, Orthopedics, Spine

## Abstract

**Purpose:**

To explore whether it is necessary to put drain tubes after posterior pedicle screw fixation of thoracolumbar fractures.

**Methods:**

From April 2020 to January 2023, a total of 291 patients with recent thoracolumbar fractures (AO type-A or type-B) who received the pedicle screw fixation operation were enrolled retrospectively. In 77 patients, drain tubes were used in the pedicle screw fixation surgery, while no drain tubes were placed in the other group. After gleaning demographic information and results of lab examination and imageology examination, all data were put into a database. Independent-sample t-tests, Pearson Chi-Square tests, Linear regression analysis, and correlation analysis were then performed.

**Results:**

Compared to the control group, the drainage group had significantly lower postoperative CRP levels (*P* = 0.047), less use of antipyretics (*P* = 0.035), higher ADL scores (*P* = 0.001), and lower NRS scores (*P* < 0.001) on the 6th day after surgery. Other investigation items, such as demographic information, operation time, intraoperative blood loss, body temperature, and other preoperative and postoperative lab results, showed no significant differences.

**Conclusions:**

The use of a drain tube in the pedicle screw fixation of thoracolumbar fractures is correlated with the improvement of patients’ living and activity ability and the reduction of inflammation, postoperative fever and pain.

## Introduction

Hematoma compression may result in a neurologic deficit; thus, drains are commonly used in spinal surgery [1]. Some researchers believe that using the drain after spinal surgery could decrease wound drainage and, as a result, decrease infection rates [2–4]. Mirzai [5] demonstrated that using a drain decreases both the incidence and size of hematoma on the first postoperative day. Mohamed et al. [6] found less epidural fibrosis and better clinical outcomes in spinal surgery with wound drains. However, there is no consensus for the use of drains. Some believe that the drains left in place for a prolonged period of time would have a higher rate of bacterial contamination than those in place for a shorter duration [7]. Chen [8] reported that drainage tube placement can reduce the infection rate after cervical surgery. Raunak [9] found that the placement of postoperative drainage tubes did not increase the incidence of postoperative complications. Many infectious disease specialists believe that the use of a drain increases the risk of infection, which outweighs the purpose of using it (i.e., decreasing hematoma risk and postoperative neurologic difficulties). The use of drains is associated with an increased prevalence of postoperative fever, which could be a reaction to the invasiveness of surgery and the nature of drains as a foreign body [10]. Some studies have shown that the use of drains does not influence the risk of wound infection and hematomas in single-level lumbar decompression surgery [11, 12]. Some systematic reviews have suggested that the routine use of a wound drain in noncomplex lumbar surgery did not prevent postoperative epidural hematomas and that the absence of a drain did not lead to a significant change in the incidence of wound infection [13, 14]. Some found a possible benefit regarding postoperative hematoma; infections and hospital stay were assumed but were not certainly proven [15]. Related complications of wound drains are also discussed in multiple ways, for example, fever, blood loss, anemia, infections or greater blood transfusion rates [16].

Is using the drain necessary in spinal surgery? This is a surgical problem that needs to be solved. Therefore, to minimize interference factors, patients with thoracolumbar fractures were treated with simple posterior pedicle screw fixation, but no decompression was chosen. We tried to find evidence indicating the superiority or inferiority of using the drain tube.

## Materials and methods

After approval from the Institutional Review Board, we retrospectively reviewed the records of thoracolumbar fractures treated with simple posterior pedicle screw fixation but no decompression between April 2020 and January 2023 at our department.

The inclusion criteria were as follows: (1) at least 18 years of age, (2) recent thoracolumbar fractures (T11 to L2, < 1 week after trauma), (3) adult single segment thoracolumbar fractures with operative indications, (4) fractures belonging to AO type-A or type-B, (5) patients who underwent pedicle screw fixation with short-segmental fixation using angular stable pedicle screw systems, (6) a posterior paraspinal muscle approach, and (7) actual treatment in compliance with the following general procedure statement.

The following patients were excluded: (1) old thoracolumbar fractures, (2) fractures with neurological deficits, (3) fractures associated with other severe injuries or vital organ damage, (4) operation associated with decompression of the spinal canal, (5) operation associated with bone grafting, (6) operation associated with vertebroplasty, (7) operation associated with fusion process, (8) patients less than 18 years old, (9) consecutive multiple segment thoracolumbar fractures with operative indications, and (10) patients who received other surgeries during hospitalization.

We reviewed the electronic medical record system, sieved patients with the above criteria, extracted demographic information, gathered lab results, and then gathered data and stored them anonymously in a database, namely, age, sex, weight, hypertension, diabetes, hepatic adipose infiltration, other fractures, operation time, intraoperative blood loss, use of drainage, postoperative hospital stay, drainage volume, number of antipyretic use, neurogenic exercise for UE(NEU), hemoglobin(Hb), platelets(PLT), C-reactive protein (CRP) tests, activities of daily living (ADL) scores, and numerical rating scale (NRS). The corresponding author checked the consistency between them.

### General treatment procedure

At our department, inpatients with AO type-A/B thoracolumbar fractures indicative for the operation were sufficiently prepared for the operation. After a rigorous preoperative examination and sufficient preoperative evaluation, the patient was placed in a prone position under general anesthesia. The skin was prepared and draped in a conventional orthopedic method, and a posterior median skin incision was made centered on the fracture segment. The skin incision was carried down to the level of the lumbodorsal fascia, and then the soft tissues were retracted laterally on either side so that longitudinal fascial incisions could be made 2 cm lateral to the median line. Once the deep fascia was dissected, the natural cleavage plane between the multifidus and longissimus muscle was exposed, and the finger could plunge into the plane and reach the facet joint. Gelpi retractors were placed between the two muscle groups. The muscle fibers attached to the deep fascia were separated from it, and the soft tissues were gently retracted with an electrotome to expose bilateral facet joints [17]. After transpedicular puncture, the two pedicle screws were separately inserted in the fracture segment, upper segment, and the nasal segment vertebral bodies with guidance by the C-arm. Then, the pedicle screw system was installed after satisfactory reduction was obtained. After hemostasis and flushing, a 16-gauge drain tube was placed before suturing. The placement and use of the drainage tube were based on the clinical experience of our surgical team at our hospital. The drain tube was positioned subfascially, and only one tube was utilized. The drain remained under partial pressure and was not emptied or changed intermittently during the observation period. In the following three days, patients were treated with cefuroxime sodium 1.5 g, iv, bid, and 3d to prevent inflammation. There was no clamping in any of the drain tubes. Drain tubes were routinely removed on the 48–52 h after surgery. The postoperative blood tests were checked on the 1st day after surgery. Patients’ postoperative body temperatures were checked every 8 h. Only when body temperature exceeded 38 °C was the patient regularly treated with an antipyretic (Indometacin suppositories, 50 mg, via rectum). Repeated antipyretic usage was dosed after 8 h according to body temperature. The number of antipyretic uses was counted as an objective sign of fever. In the review, patients receiving any treatment against this procedure were excluded.

### Statistical analysis

Gathered data were sorted and classified with WPS Version Pro, and then statistical evaluations were carried out using SPSS Statistics Version 22. We chose independent-sample t tests to measure continuous normally distributed variables and chi-square tests to measure binary variables. For the drainage volume in the drainage group, correlation tests and multiple linear regression tests were performed. Variables with a *P* value less than 0.05 were regarded as significant [18].

## Results

In accordance with the inclusion criteria and exclusion criteria, a total of 291 patients (45.31 ± 11.08 years, 181 male and 110 female) with AO type-A or type-B thoracolumbar fractures who received pedicle screw fixation by the posterior paraspinal muscle approach were enrolled in our study. All patients suffered from single segment fractures with operative indications, 11 patients with T11 fractures, 54 with T12 fractures, 148 with L1 fractures, and 78 with L2 fractures. A total of 43 patients had other recent fractures, including 12 patients with upper limb fractures, 15 patients with lower limb fractures, 4 patients with other spine fractures, 9 with rib fractures, and 3 with pelvic fracture. All these associated fractures were treated with conservative treatment. None of these patients had fever or flu-like symptoms before surgery. Based on the usage of drainage tubes, we retrospectively divided these patients into the drainage group and the control group, with 77 patients in the drainage group and 214 patients in the control group. Other demographic and medical information data are also displayed in Table 1.

**Table 1:** Comparison of general and medical conditions of the two groups Significant differences between the drainage and control groups are indicated as * *P* < 0.05, ** *P* < 0.01, *** *P* < 0.001

	Control group	Drainage group	t or χ2	***P value***	Significance
N	214	77			
Gender (n)					
Male	136	45	0.63	0.428	
Female	78	32			
Age (year)	44.83 ± 11.3	46.64 ± 10.42	-1.23	0.220	
Weight (kg)	65.23 ± 9.98	64.06 ± 11.94	0.77	0.446	
Intraoperative blood loss (ml)	121.18 ± 53.47	120.32 ± 49.71	0.12	0.903	
Operation time (min)	84.87 ± 14.6	85.83 ± 13.66	-0.50	0.616	
Postoperative hospital stay (day)	7.14 ± 1.99	7.25 ± 1.46	-0.45	0.654	
Drainage volume (ml)	—	76.10 ± 34.56			
NEU (×109/L)					
Preoperative	5.58 ± 1.9	5.94 ± 2.03	-1.42	0.158	
Postoperative	7.60 ± 2.39	7.39 ± 2.19	0.67	0.502	
Hb (g/L)					
Preoperative	138.99 ± 15.34	138.17 ± 12.93	0.42	0.676	
Postoperative	127.42 ± 15.21	128.4 ± 28.32	-0.38	0.704	
PLT (×109/L)					
Preoperative	210.67 ± 61.63	208.09 ± 47.58	0.38	0.708	
Postoperative	216.52 ± 62.48	217.95 ± 53.61	-0.18	0.859	
CRP (mg/L)					
Preoperative	17.71 ± 13.71	20.38 ± 18.55	-1.15	0.251	
Postoperative	53.4 ± 35.69	44.38 ± 28.73	2.00	0.047	*
ADL score					
Preoperative	35.44 ± 8.59	34.52 ± 13.66	0.55	0.582	
The 2nd postoperative day	34.39 ± 9.82	33.03 ± 10.26	1.04	0.301	
The 6th postoperative day	46.3 ± 11.60	50.99 ± 9.54	-3.49	0.001	***
NRS score					
Preoperative	5.20 ± 1.65	5.27 ± 1.40	-0.34	0.734	
The 2nd postoperative day	4.66 ± 1.57	4.60 ± 1.55	0.32	0.750	
The 6th postoperative day	4.40 ± 1.30	2.90 ± 1.14	9.01	< 0.001	***
Hypertension (n)	41	21	2.22	0.136	
Diabetes (n)	28	9	0.10	0.753	
Hepatic adipose infiltration (n)	67	16	3.08	0.079	
Other fractures (n)	35	8	1.60	0.206	
Number of antipyretic use (n)					
Once	46	8	8.61	0.035	*
Twice	19	3			
Third	6	1			

Significant differences between the drainage and control groups are indicated as * *P* < 0.05, ** *P* < 0.01, *** *P* < 0.001

Furthermore, we analyzed all the possible factors for actual operation by independent-sample t test (Table 1). There were no significant differences in demographic information (gender, age, weight, hypertension, diabetes, hepatic adipose infiltration, associated with other fractures) between the two groups. Furthermore, no infection or any other complications were observed during hospitalization.

**Fig. 1:**
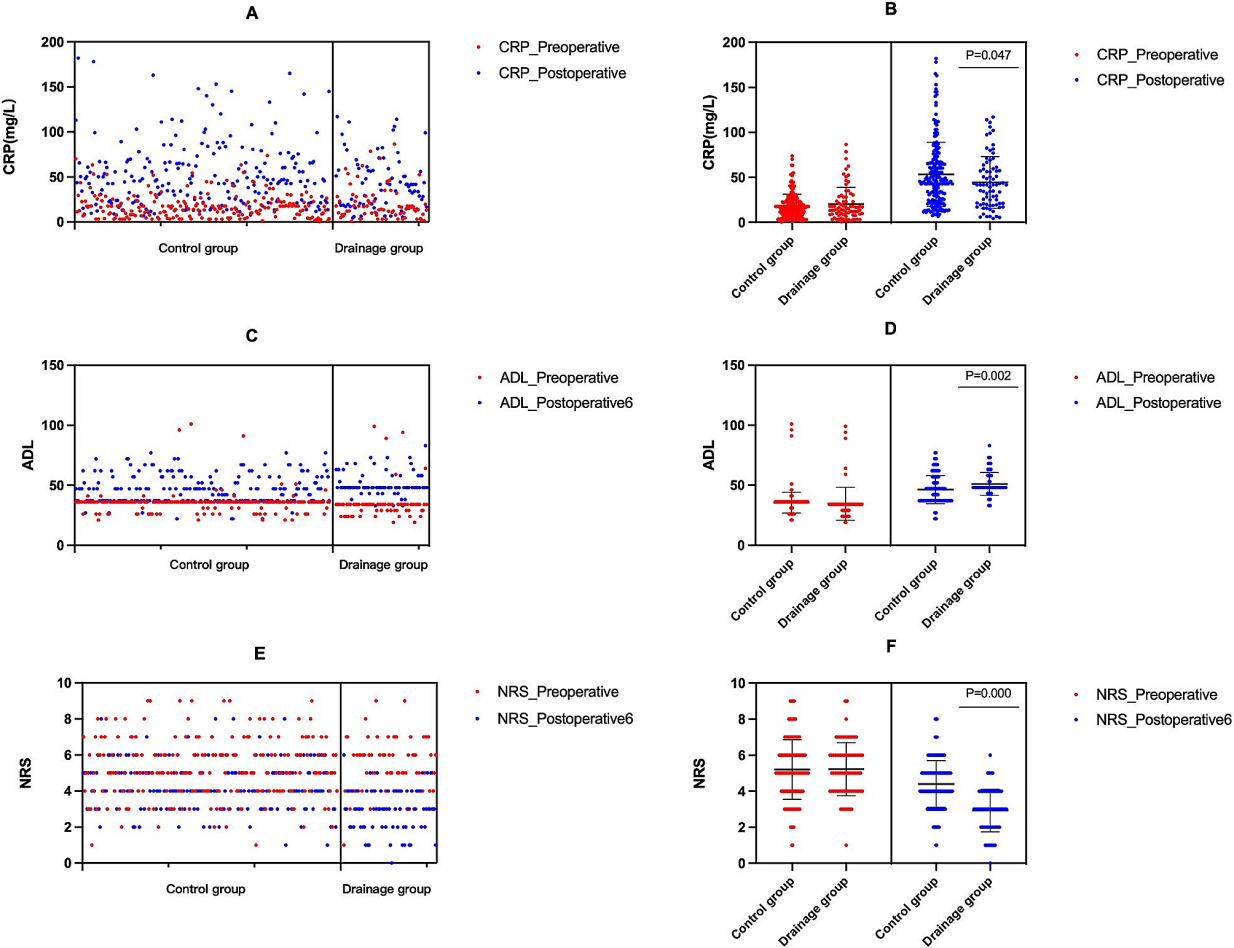
**A**: The scatter plot of CRP in both the drainage and control groups; **B**: The significant differences in CRP between the drainage and control groups; **C**: The scatter plot of ADL in both the drainage and control groups; **D**: The significant differences in ADL between the drainage and control groups; **E**: The scatter plot of NRS in both the drainage and control groups; **F**: The significant differences in NRS between the drainage and control groups

As shown in Table 1, there were no significant differences between the two groups in operation time, intraoperative blood loss, preoperative and postoperative NEU, Hb, PLT, length of postoperative hospital stay, preoperative CRP, ADL score, NRS score or so on. Only postoperative CRP (*P* = 0.047), the 6th postoperative day’s ADL (*P* = 0.001), and the 6th postoperative day’s NRS (*P* < 0.001) were found to be statistically significant. Compared to the control group, the drainage group had a lower level of CRP and lower NRS and ADL scores on the 6th day after surgery. The significant differences in CRP, ADL and NRS scores between the drainage group and the control group are shown in Fig. 1.

Dichotomous data were analyzed by Pearson chi-square tests, and the results are shown in Table 1. Among all these items, only the *P* value for antipyretic use was less than 0.05.

The four significant items were then tested by ANOVA (Table 2). According to the results, the 6th postoperative day’ NRS (p = 0.000), the 6th postoperative day’ ADL (*p* = 0.004), the number of antipyretic uses (*p* = 0.013), and postoperative CRP (*p* = 0.037) were detected with significance.

**Table 2:** The correlation between drainage tube placement and four items

	The 6th postoperative day’ NRS		The 6th postoperative day’ ADL		Number of antipyretic use		Postoperative CRP	
	F	***P***	F	***P***	F	***P***	F	***P***
Drainage	82.935	0.000***	8.650	0.004**	6.258	0.013*	4.393	0.037*
Age	0.883	0.348	0.001	0.971	0.106	0.745	0.096	0.757
Gender	0.199	0.656	0.029	0.864	2.425	0.121	0.916	0.339
Weight	1.105	0.294	0.738	0.391	1.690	0.195	3.137	0.078
Hypertension	2.254	0.134	4.040	0.045*	0.819	0.366	1.016	0.314
Diabetes	0.379	0.539	0.235	0.628	0.856	0.356	0.043	0.836

Significant differences are indicated as * *P* < 0.05, ** *P* < 0.01, *** *P* < 0.001

For the drainage group, linear model regression was conducted to investigate the correlation between drainage volume and other items. However, the R squared was only 0.139, suggesting that this model was not truly appropriate (Table 3).

**Table 3:** The correlation between drainage volume and other items

	Coefficients	SE	t	***P*** value
Age	0.178	0.431	1.371	0.175
Weight	-0.002	0.347	-0.017	0.986
Operation time	0.103	0.373	0.699	0.487
Intraoperative blood loss	-0.027	0.088	-0.214	0.831
Postoperative hospital stay	-0.29	3.094	-2.219	0.030*
Postoperative NEU	-0.104	1.984	-0.826	0.412
Postoperative Hb	0.034	0.153	0.27	0.788
Postoperative PLT	-0.136	0.085	-1.037	0.304
Postoperative CRP	0.064	0.156	0.491	0.625
The 6th postoperative day’ ADL	-0.102	0.547	-0.677	0.501
The 6th postoperative day’ NRS	-0.015	3.835	-0.115	0.909
Number of antipyretic use	0.063	7.885	0.48	0.633

Significant differences are indicated as * *P* < 0.05

## Discussion

In spinal surgery, drainage is generally used in clinical practice [19, 20]. The use of drainage is mainly supposed to reduce the accumulation of blood in the subfascial or epidural space and decrease the risk of spinal cord compression, neurologic deficit or infection [21, 22]. Although there have been some research papers [23], there is no striking evidence indicating the superiority or inferiority of using drain tubes in posterior spinal surgery to date. To minimize interference factors, we retrospectively chose patients with thoracolumbar fractures who underwent pedicle screw fixation with short-segment fixation using angular stable pedicle screw systems as our study subjects. To our knowledge, this is the first study to clarify whether it is necessary to place drain tubes after pedicle screw fixation.

No difference was found in all preoperative parameters, and they were, in fact, comparable, even though we grouped retrospectively. It seemed to confirm that because of no theoretical support, the use of drain tubes relies on doctors’ preference [24]. No deep infection or other serious complications occurred during hospitalization. In all laboratory results, only postoperative CRP was found to be significantly different (*P* = 0.047), while neutrophil counts showed no significant difference. These findings might indicate that the use of drainage tubes could reduce inflammatory reactions after pedicle screw fixation, which is in line with the former conclusion that drainage could reduce the risk of infection [2, 3].

ADL meant living and activity capacity, measures of the basic activities of daily living, mobility, and instrumental activities of daily living [25]. After posterior pedicle screw fixation, the ADL score showed no difference on the 2nd day, but a statistically significant difference was found on the 6th day (ADL: *P* = 0.001). Similarly, the NRS score on the 6th day was significantly lower in the drainage group (NRS: *P* < 0.001). The NRS score, which reflected the intensity of pain [26], indicated that placement of a drain tube was negatively correlated with postoperative pain. In other words, in patients after pedicle screw fixation, on the 6th postoperative day after the drain tube was removed, the use of the drain tube was associated with improved mobility and alleviated pain. Our results were completely different from those of drainage after laparoscopic cholecystectomy or thyroidectomy [27, 28], and their studies demonstrated that inserting a drain tube intensified postoperative pain. This may be related to the degree of trauma or the placement of the drain tube.

Furthermore, our results showed that there were significant differences in the number of antipyretic uses between the drainage group and the control group. The use of antipyretics, as a strong indicator of postoperative fever, suggested that the use of drainage tubes was related to a reduction in postoperative fever. This finding seemed to be consistent with the findings of other researchers, such as Brown et al. [29], who found that patients without drainage had a higher temperature than patients with drainage on the first day after surgery. Fever does not always indicate infection, and many studies [10, 13, 30] have suggested that the use of a drain in lumbar spine surgery does not lead to a significant change in the incidence of wound infection. The risk of postoperative fever in the drainage group was lower, which may be due to the decrease in the incidence and size of spinal epidural hematoma [31]. Mohamed et al. [6] also concluded that implantation of closed-suction drainage resulted in less formation of epidural fibrosis in patients operated on for unilateral, single-level lumbar disc hernias. Previous studies reported that multilevel procedures, deficient coagulation factors, decreased hemoglobin, advanced age, excessive drinking and previous spinal surgery were identified as risk factors for developing a postoperative epidural hematoma [32–35]. Therefore, these factors should be considered when deciding whether to use a drain tube after pedicle screw fixation.

It cannot be denied that there are some limitations to our study. First and foremost, it was regrettable that we did not gather long-term follow-up and functional result data. Second, our study is based on a single center, and our sample size might not be large enough to reveal some hidden correlations. Third, the retrospective nature of the study might introduce some bias. Even so, our study results might reveal that patients in the drainage group had less systemic inflammation, less pain, and better viability after posterior pedicle screw fixation. More well-designed, large-sample randomized controlled subsequent studies are required to further explain the value of drain tubes in pedicle screw fixation.

## Conclusion

In conclusion, the use of drain tubes in the pedicle screw fixation of thoracolumbar fractures is correlated with the improvement of patients’ living and activity ability and the reduction of inflammation, postoperative fever and pain. Of course, more follow-up studies are needed to explain the additional value of drainage tubes in pedicle screw fixation and to explain why they reduce inflammation, postoperative fever, and pain.

## Data Availability

The datasets generated and analyzed during the current study are available from the corresponding author on reasonable request.

## Abbreviations

CRP: C-reactive protein
ADL: Activities of Daily Living
NRS: Numerical Rating Scale
NEU: Neutrophil count
HB: Hemoglobin
PLT: Platelet count
